# Perceptions of provider awareness of traditional and cultural treatments among Indigenous people who use unregulated drugs in Vancouver, Canada

**DOI:** 10.1186/s12913-024-10672-2

**Published:** 2024-03-02

**Authors:** Alexa Norton, Fahmida Homayra, Courtney Defriend, Brittany Barker, Louise Meilleur, Kanna Hayashi, Bohdan Nosyk

**Affiliations:** 1 Centre for Advancing Health Outcomes, Vancouver, BC Canada; 2 BC Centre on Substance Use, Vancouver, BC Canada; 3 Faculty of Health Sciences, Simon Fraser University, Burnaby, BC Canada; 4 First Nations Health Authority, West Vancouver, BC Canada; 5 Interdisciplinary Studies Graduate Program, University of British Columbia, Vancouver, BC Canada

**Keywords:** Cultural safety, Primary care, Barriers to care, Indigenous Peoples, People who use drugs, Traditional and cultural treatments, Indigenous cultural safety training

## Abstract

**Introduction:**

Indigenous people who use unregulated drugs (IPWUD) face significant barriers to care, including sparse availability of culturally safe health services. Integrating Indigenous traditional and cultural treatments (TCT) into health service delivery is one way to enhance culturally safe care. In a Canadian setting that implemented cultural safety reforms, we sought to examine the prevalence and correlates of client perceptions of primary care provider awareness of TCT among IPWUD.

**Methods:**

Data were derived from two prospective cohort studies of PWUD in Vancouver, Canada between December 2017 and March 2020. A generalized linear mixed model with logit-link function was used to identify longitudinal factors associated with perceived provider awareness of TCT.

**Results:**

Among a sample of 507 IPWUD who provided 1200 survey responses, a majority (*n* = 285, 56%) reported their primary care provider was aware of TCT. In multiple regression analyses, involvement in treatment decisions always (Adjusted Odds Ratio [AOR] = 3.6; 95% confidence interval [CI]: 1.6–7.8), involvement in treatment decisions most or some of the time (AOR = 3.3; 95% CI: 1.4–7.7), comfort with provider or clinic (AOR = 2.7; 95% CI: 1.5–5.0), and receiving care from a social support worker (AOR = 1.5; 95% CI: 1.0–2.1) were positively associated with provider awareness of TCT.

**Conclusion:**

We found high levels of perceived provider awareness of TCT and other domains of culturally safe care within a cohort of urban IPWUD. However, targeted initiatives that advance culturally safe care are required to improve healthcare and health outcomes for IPWUD, who continue to bear a disproportionate burden of substance use harms.

## Introduction

People who use unregulated drugs (PWUD) often encounter challenges accessing and receiving quality health services. In this paper, we use the term ‘unregulated’ to describe drugs that are of unknown composition and concentration and are available through underground markets and street economies [1]. Stigma, discrimination, and a range of barriers related to access may discourage or prevent PWUD from seeking care, resulting in high rates of perceived unmet health needs [2, 3]. These challenges are worrisome, given that PWUD often have concurrent health needs related to substance use, including mental health and wellness and the prevention and management of infectious diseases [4]. Indigenous people in Canada who use drugs (IPWUD) face additional barriers to care, including intersectional stigma and discrimination and sparse availability of culturally safe health services [5–7]. As health system interventions are implemented to advance cultural safety, measurement is imperative to understand ongoing patterns of institutionalized anti-Indigenous racism and the dimensions of culturally safe and equitable care [8].

In recent years, there has been movement to implement cultural safety throughout health systems in Canada and globally [9, 10]. Cultural safety is a concept and practice that aims to address power imbalances inherent in healthcare systems and at the point of care [11–13]. In Canada, strategies for implementation have included Indigenous cultural safety policies, the creation of Indigenous health authorities, such as the provincial First Nations Health Authority (FNHA) in British Columbia (BC), health systems navigation supports (e.g., Indigenous Patient Navigators), and Indigenous cultural safety training programs and core competencies for health professionals. This movement toward cultural safety aims to dismantle racist healthcare structures that have deterred Indigenous people from accessing and receiving quality care.

As the movement toward cultural safety accelerates, there is a need for client-defined outcomes of culturally safe care. There is a small body of evidence from within Canada that explores providers’ cultural safety from the perspective of clients, including in midwifery [14], mainstream healthcare [15], and hospital and emergency department settings [16, 17], as well as in research with PWUD [18]. Such findings suggest that respect, trust, safety, and kindness are central in clinical encounters, which should be guided by adherence to professional standards, accommodation of needs and circumstances, clear communication, affirmation of personal and cultural identities, shared decision-making, and the offering of culturally safe practices and spaces. While a toolkit for evaluating cultural safety within health systems exists in BC [19], no measures of cultural safety, client-defined or otherwise, are yet reported nationally [8].

One critical component of cultural safety involves integrating Indigenous traditional and cultural treatments into service delivery [20]. Such treatments include those offered within conventional medical care (e.g., physical spaces in health facilities for cultural protocol and ceremony, Elder care and spiritual advising, traditional medicines and foods) as well as those offered outside of conventional care settings (e.g., land-based healing initiatives, sweat lodge ceremonies, Indigenous treatment centres) [21, 22]. Globally, integrated traditional and cultural treatments have been shown to support Indigenous people living with cancer, dementia, addictions, and diabetes to support their mental, emotional, and spiritual wellness [20, 23–26]. In Canada, Indigenous people underutilize health services, and mental health and addictions services in particular, due to their lack of cultural safety and relevancy [27, 28]. This perpetuates illness in a cyclical fashion wherein Indigenous people do not feel safe to access healthcare, accessing only when there is dire need, which reinforces racist stereotypes in care settings, further deterring non-acute access [5]. Raising awareness of and integrating traditional and cultural treatments into health service delivery may be one pathway to enhanced access and quality for IPWUD.

In 2016, the regional Vancouver Coastal Health (VCH) authority in BC, Canada, launched its Downtown Eastside Second Generation Strategy (DTES-2GS), a multi-year healthcare system reform [29]. The Downtown Eastside (DTES) is a neighbourhood in Vancouver often characterized by its visible structural marginalization and high rates of substance use and poverty [30, 31]. This is in part due to its history of colonial dispossession [32]. The DTES has a high concentration of Indigenous residents [33] who have long reported formidable barriers to healthcare [34, 35]. However, it is also a community with a history of resilience and activism that has created and sustained health and social services for Indigenous residents, PWUD, and other groups [36–39]. The DTES-2GS involved the implementation of a new model of care, including integrated care teams and interdisciplinary primary care clinics which offer primary care, mental health and substance use services, harm reduction, Elder care, and specialized care (e.g., wound care, palliative care, occupational therapy) in one location [29]. Engagement with Indigenous stakeholders and clients enhanced the new model of care, improving supports for Indigenous clients [40, 41]. Initiatives such as a mandatory Indigenous cultural safety core competency for health staff; coordinated service planning with Indigenous stakeholders; and the provision of Indigenous cultural services, including an Elders-in-residence program, Indigenous-specific naloxone distribution sites and training, and Indigenous design concepts in renovations and new builds fostered awareness of Indigenous traditional and cultural treatments among healthcare providers [29]. Thus, we sought to examine the prevalence and longitudinal correlates of perceptions of primary care provider awareness of traditional and cultural treatments among IPWUD.

## Methods

### Data sources

This analysis uses data from the DTES-2GS evaluation (hereafter referred to as the “2GS Supplement”), a linked prospective cohort study designed to assess changes in healthcare access and quality resulting from the implementation of the DTES-2GS. Participants were recruited from two ongoing prospective cohort studies of PWUD in Vancouver, Canada: the Vancouver Injection Drug Users Study (VIDUS) and the AIDS Care Cohort to Evaluate exposure to Survival Services (ACCESS). Both cohorts have been described in detail elsewhere [42, 43]. VIDUS enrolls HIV-negative adults who report injecting an unregulated drug at least once in the month prior to enrolment; ACCESS is a cohort of adults living with HIV who report using an unregulated drug (other than or in addition to cannabis) in the month prior to enrolment. The cohorts have been recruited through snowball sampling, street outreach, and self-referral since 1996 (VIDUS) and 2005 (ACCESS). At baseline and semi-annually, participants complete an interviewer-administered questionnaire that gathers sociodemographic data and information about substance use patterns, health and social behaviours, and healthcare utilization. Study instruments and follow-up procedures are harmonized to enable combined analyses. Written informed consent is obtained from participants who receive a $40 (CAN) honorarium for each study visit.

### Study population and questionnaire development

Between December 2017 and October 2019, VIDUS and ACCESS participants were invited to participate in the 2GS Supplement. Given the parent cohorts’ community recruitment methods, 1004 participants were recruited through convenience sampling. Data were collected using an interviewer-administered questionnaire developed in partnership with three peer-led community organizations of PWUD, including one Indigenous peer group, the Western Aboriginal Harm Reduction Society (WAHRS) [44], with questions adapted from the World Health Organization (WHO) Survey on Health and Health System Responsiveness [45] The questionnaire collected information on healthcare access, prompt attention (timeliness), dignity and respect, communication, autonomy, confidentiality, choice, quality, outpatient facility evaluation, and cultural safety. The section on cultural safety was not adapted from the WHO survey but developed by the study team in partnership with WAHRS and BC’s FNHA to observe whether the implementation of the DTES-2GS would correspond to changes in healthcare access and quality for IPWUD. 2GS data collection was scheduled on the same day as VIDUS and ACCESS follow-up appointments or within two weeks if participants were unable to participate on the same day. Semi-annual follow-up questionnaires were completed at a maximum of four times, with data collection for the 2GS Supplement ending in March 2020. Participants provided their consent verbally and received an additional $15 (CAD) to complete the 2GS Supplement questionnaire. The University of British Columbia/Providence Healthcare Research Ethics Board provided ethical approval for all studies.

### Measures and outcomes

The current study used baseline and follow-up data from the 2GS Supplement and its linked parent cohorts. Our analysis is restricted to participants who completed a questionnaire between December 2017 and March 2020, self-identified as Indigenous (First Nations, Métis, and/or Inuit), and reported accessing healthcare in the last 6 months. It is further restricted to participants who answered the question from the cultural safety section of the questionnaire, “Do you feel that your provider is aware of any traditional or culturally based alternatives to treatment? *For example, contact with a spiritual care provider or Elder, access to traditional medicines or medical practices, methods of land-based healing?”* The primary outcome of interest was a binary measure (yes vs. no) of perceptions of provider awareness of traditional and cultural treatments. Explanatory variables of interest were selected based on hypothesized relationships using the Anderson (1995) [46] healthcare utilization model, which distinguishes between predisposing characteristics, enabling resources, and need factors that shape individuals’ use of health services. We considered the predisposing factors of age, stratified into four categories (25–35, 36–45, 46–55, >55), self-identified gender (man vs. woman vs. gender minorities [Two-Spirit, trans man, trans woman, other]), homelessness/unstable housing (yes vs. no), and residence in the DTES neighbourhood (yes vs. no). Enabling factors included patient involvement in treatment decisions, stratified by frequency (always vs. most or some of the time vs. never), comfort with primary care provider or clinic (yes vs. no or not having a regular family doctor or clinic to go to), whether most required healthcare was received in one place (yes vs. no), and type of provider that care was received from most frequently, stratified by training and type (clinician [doctor, methadone-only doctor, nurse, nurse practitioner, psychiatrist, pharmacist] vs. social support worker [counsellor, outreach worker, social worker, peer]). Need factors included chronic pain (yes vs. no), defined as reporting pain that has persisted for greater than 3 months [47], a concurrent mental health condition (yes vs. no) for which care was needed or accessed (e.g., anxiety/panic, depression, mood disorder), delayed care due to past poor treatment (yes vs. no), delayed treatment due to racism (yes vs. no), and healthcare refused due to drug use (yes vs. no). All variables were measured in the previous 6 months. We included the survey round as an explanatory variable to control for time trend as well as the cohort designation (VIDUS vs. ACCESS).

### Statistical analyses

First, we examined the prevalence of each explanatory variable at baseline, stratified by perceptions of provider awareness of traditional and cultural treatments (yes vs. no). We then fit a multiple logistic regression model with the primary outcome as client perceptions of provider awareness of traditional and cultural treatments and explanatory variables capturing predisposing characteristics, enabling resources, and need factors described above. We used a strengths-based approach in identifying factors associated with provider awareness of culturally safe care in order to reinforce positive change [48]. We applied a generalized linear mixed effects model with logit-link function assuming heterogeneity across study participants, and we incorporated a random intercept at participant level to adjust for within-individual correlation resulting from longitudinal repeated measures [49]. All *p*-values were two-sided and statistical analyses conducted using SAS 9.4.

## Results

Between December 2017 and March 2020, 507 participants self-identified as Indigenous (First Nations, Métis, and/or Inuit). In total, there were 1200 survey responses with a median of 2 (quartile [Q]1=1, Q3=3) surveys conducted per participant. At baseline, 252 (49.7%) participants identified as women and 14 (2.8%) identified as gender minorities (Two-Spirit, trans man, trans woman, other). Among all participants, 285 (56.2%) reported “yes” to provider awareness of traditional and cultural treatments. A majority reported they were comfortable with their primary care provider or clinic (450, 88.8%), received their healthcare in one place (414, 81.7%), and received care most frequently from a clinician rather than a social support worker (386, 76.1%). A majority lived in the DTES (349, 68.8%), were experiencing unstable housing or homelessness (334, 65.9%), and were experiencing chronic pain (299, 59%) (Table 1).

**Table 1:** Baseline predisposing, enabling, and need factors stratified by client perceptions of provider awareness of traditional and cultural treatments among Indigenous people who use unregulated drugs (*n* = 507) in Vancouver, Canada, from December 2017 to March 2020

	**Client perceptions of provider awareness of traditional and cultural treatments**		
**Characteristic**	No (%) *n* = 222 (43.8%)	Yes (%) *n* = 285 (56.2%)	Total *n* = 507 (100%)
*Pre-disposing factors*			
Age^a^			
25 – 35	25 (4.9)	44 (8.7)	69 (13.6)
35 – 45	69 (13.6)	75 (14.8)	144 (28.4)
45 – 55	76 (15.0)	104 (20.5)	180 (35.5)
>55	52 (10.3)	62 (12.2)	114 (22.5)
Self-identified gender^a^			
Woman	120 (23.7)	132 (26.0)	252 (49.7)
Man	96 (18.9)	138 (27.2)	234 (46.2)
Gender minorities^b^	4 (0.8)	10 (2.0)	14 (2.8)
Unstable housing/homelessness^a^	143 (28.2)	191 (37.7)	334 (65.9)
DTES residence^a^	153 (30.2)	196 (38.7)	349 (68.8)
*Enabling factors*			
Patient involvement in treatment decisions^a^			
Always	89 (17.6)	138 (27.2)	227 (44.8)
Most or some of the time	28 (5.5)	28 (5.5)	56 (11.0)
Never	20 (3.9)	5 (1.0)	25 (4.9)
Comfort with provider or clinic^a^	190 (37.5)	260 (51.3)	450 (88.8)
Received healthcare in one place^a^	179 (35.3)	235 (46.4)	414 (81.7)
Provider type^a^			
Clinician	177 (34.9)	209 (41.2)	386 (76.1)
Social support worker	45 (8.9)	76 (15.0)	121 (23.9)
*Need factors*			
Chronic pain^a^	127 (25.0)	172 (33.9)	299 (59.0)
Mental health^a^	78 (15.4)	85 (16.8)	163 (32.1)
Barriers to care^a^			
Delayed care due to poor treatment	56 (11.0)	59 (11.6)	115 (22.7)
Delayed treatment due to racism	27 (5.3)	29 (5.7)	56 (11.0)
Refused healthcare due to drug use	45 (8.9)	39 (7.7)	84 (16.6)
Parent cohort			
VIDUS	123 (55.4)	155 (54.4)	278 (54.8)
ACCESS	99 (44.6)	130 (45.6)	229 (45.2)

*DTES* Downtown Eastside, *VIDUS* Vancouver Injection Drug Users Study, *ACCESS* AIDS Care Cohort to Evaluate Exposure to Survival Services. All yes/no variables are presented in the affirmative

^a^Denotes behaviours and events in the previous 6 months

^b^Inclusive of Two-Spirit, trans man, trans woman, and other gender

Compared to those who did not perceive provider awareness, those reporting provider awareness of traditional and cultural treatments reported always being involved in treatment decisions (27.2% vs. 17.6%), comfort with their provider or clinic (51.3% vs. 37.5%), and receiving care more frequently from clinicians (41.2% vs. 34.9%) and social support workers (15% vs. 8.9%). Those reporting provider awareness also reported more unstable housing or homelessness (37.7% vs. 28.2%) and chronic pain (33.9% vs. 25%) (Table 1).

In multiple regression analyses (Table 2), factors that were positively associated with perceived provider awareness of traditional and cultural treatments included always being involved in treatment decisions (Adjusted Odds Ratio [AOR] = 3.6; 95% confidence interval [CI]: 1.6–7.8), being involved in treatment decisions most or some of the time (AOR = 3.3; 95% CI: 1.4–7.7), comfort with provider or clinic (AOR = 2.7; 95% CI: 1.5–5.0), and receiving care from a social support worker (AOR = 1.5; 95% CI: 1.0–2.1). Awareness also improved over time: the adjusted odds of awareness were higher in the fourth and final survey round (AOR = 2.5; 95% CI: 1.0–6.1) compared to the baseline survey.

**Table 2:** Multiple logistic regression analyses of predisposing, enabling, and need factors associated with client perceptions of provider awareness of traditional and cultural treatments among Indigenous people who use unregulated drugs (*n* = 507) in Vancouver, Canada, from December 2017 to March 2020

**Characteristic**	**Unadjusted odds ratio (95% CI)**	***p-*value**	**Adjusted Odds Ratio (95% CI)**	***p*-value**
*Pre-disposing factors*				
Age^**a**^				
36–45 vs. 25–35	0.9 (0.5, 1.6)	0.67	0.8 (0.5, 1.4)	0.47
46–55 vs. 25–35	1.2 (0.7, 2.0)	0.60	1.0 (0.6, 1.8)	0.92
>55 vs. 25–35	1.0 (0.5, 1.7)	0.86	0.8 (0.4, 1.5)	0.47
Self-identified gender^**a**^				
Man vs. woman	1.2 (0.9, 1.7)	0.22	1.2 (0.9, 1.7)	0.23
Unstable housing/homelessness^**a**^ (yes vs. no)	1.2 (0.9, 1.6)	0.32	1.2 (0.9, 1.8)	0.24
DTES residence^**a**^ (yes vs. no)	1.0 (0.8, 1.4)	0.80	1.0 (0.7, 1.5)	0.95
*Enabling factors*				
Patient involvement in treatment decisions^**a**^				
Always vs. never	5.1 (2.4, 10.8)	<0.0001	3.6 (1.6, 7.8)	0.002
Most or some of the time vs. never	4.0 (1.7, 9.0)	0.001	3.3 (1.4, 7.7)	0.01
Comfort with provider or clinic^**a**^ (yes vs. no)	3.7 (2.1, 6.6)	<0.0001	2.7 (1.5, 5.0)	0.002
Received healthcare in one place^**a**^ (yes vs. no)	1.3 (0.8, 2.0)	0.30	0.9 (0.6, 1.6)	0.80
Provider type^**a**^				
Social support worker vs. clinician	1.5 (1.1, 2.2)	0.01	1.5 (1.0, 2.1)	0.03
*Need factors*				
Chronic pain^**a**^ (yes vs. no)	1.0 (0.8, 1.4)	0.87	1.2 (0.8, 1.6)	0.36
Mental health^**a**^ (yes vs. no)	0.8 (0.6, 1.1)	0.21	0.9 (0.6, 1.3)	0.52
Barriers to care^**a**^ (yes vs. no)				
Delayed care due to poor treatment	0.6 (0.4, 0.9)	0.01	0.7 (0.5, 1.1)	0.15
Delayed treatment due to racism	0.8 (0.4, 1.3)	0.27	1.4 (0.8, 2.5)	0.29
Refused healthcare due to drug use	0.6 (0.4, 0.9)	0.01	0.7 (0.4, 1.1)	0.15
*Survey round*				
Follow-up 1 vs. baseline	1.6 (1.1, 2.3)	0.01	1.6 (1.1, 2.3)	0.01
Follow-up 2 vs. baseline	1.2 (0.8, 1.7)	0.43	1.1 (0.7, 1.5)	0.78
Follow-up 3 vs. baseline	1.3 (0.8, 2.0)	0.24	1.2 (0.8, 1.9)	0.38
Follow-up 4 vs. baseline	3.0 (1.2, 7.2)	0.02	2.5 (1.0, 6.1)	0.05

*CI* Confidence interval, *DTES* Downtown Eastside

^a^Denotes behaviours and events in the previous 6 months

## Discussion

This is the first study, to our knowledge, to characterize perceptions of provider awareness of traditional and cultural treatments among a sample of Indigenous people who use drugs (IPWUD). We observed that a majority of self-identified IPWUD (56%) felt their provider was aware of traditional and cultural treatments. Controlling for factors capturing predisposing characteristics and underlying healthcare needs, these individuals were more likely to be involved in treatment decisions and to experience comfort with their provider or clinic than those who did not report perceived awareness. We also observed that while they received care most frequently from clinicians, receiving care from a social support worker was positively associated with perceived awareness.

During 2GS Supplement questionnaire development, IPWUD reported that institutional barriers were leading to the exclusion of Indigenous cultural practices and medicines in healthcare settings [44]. In addition to these reported barriers, Indigenous people face difficulties with providers’ communication, limited institutional offerings, and providers’ limited knowledge and wariness to ‘prescribe’ them traditional and cultural treatments [50, 51]. Therefore, our finding of 56% perceived awareness is notable. Cultural awareness is integral to the implementation of cultural safety [52]. In particular, cultural awareness is critical in the context of healthcare for IPWUD, who not only underutilize mainstream health services due to their lack of cultural safety and relevancy [27, 28], but have been shown to benefit from blended Indigenous and Western approaches to health and substance use care [25, 53, 54]. While our study measured a positive change in perceived provider awareness over time, this finding must be interpreted with caution given loss of participants over time to follow-up. However, the 56% rate indicates confidence among some IPWUD that their provider is knowledgeable of Indigenous approaches to treatment and healing, which has been repeatedly identified as a critical step in reducing barriers to care [55, 44].

Given that 44% of participants reported their provider was not aware of such treatments, there is also room for improvement. Healthcare providers must have core competencies including knowledge, self-awareness, and skills that are often learned through Indigenous cultural safety education and training programs [56, 57] mandated at policy and institutional levels [6]. In 2018, Vancouver Coastal Health implemented a health authority-wide Indigenous cultural safety policy, recommending Indigenous cultural safety training for staff [58]. In addition to broad Indigenous cultural safety policies and training programs, the First Nations Health Authority recommended undertaking specific activities to continue driving change, for example, integrating cultural safety and humility skill development and assessment into job performance evaluations [59]. Awareness on its own cannot be translated into improved care; it is only one measure among many (e.g., availability and retention of healthcare providers, physical availability of healthcare services) to assess healthcare access and quality for Indigenous people [60]. Ongoing monitoring and quality improvement initiatives are required to ensure cultural safety enhancements are meeting their stated objectives, in the DTES and elsewhere, as well as identify gaps in implementation and specific ideas for change from IPWUD themselves.

Among 507 IPWUD in Vancouver, BC, a significant majority felt comfort with their provider or clinic (89%) and received their healthcare in one place (82%). A prior analysis with these data found an 84% attachment rate among a sample of PWUD in the DTES (described as having a regular doctor or clinic that they felt comfortable going to) [3]. That rates of attachment and comfort among IPWUD are so high in a neighbourhood with challenges regarding discrimination in healthcare settings is remarkable, given the inequitable access to primary care that Indigenous Peoples frequently experience, often leading to emergency department usage as a last resort [5]. In the DTES, IPWUD, and women in particular, have long described poor experiences of care and discrimination in healthcare settings based on their Indigeneity, drug use, chronic pain needs, and residence in the neighbourhood, as well as a lack of access to culturally safe health services [34, 61, 38, 35, 44]. During the development of the 2GS Supplement questionnaire, non-Indigenous and IPWUD in the DTES stated that meaningful facilitators to care included continuity of care with a trusted healthcare provider and being able to access care in one place [44]. In this respect, our findings of high rates of attachment and comfort with the provider or clinic are promising. They indicate that certain elements of the healthcare experience may be aligned with the stated desires of IPWUD in the DTES, although it is not clear whether they are the result of DTES-2GS reforms [29]. Our findings are consistent with international evidence showing integrated care improves perceived quality of care, patient satisfaction, and access to care [62]. It is worth noting, however, that even when PWUD report high levels of attachment, they may have unmet health needs. Our study found a high rate of chronic pain (59%) among IPWUD. This difference may reflect a higher burden of chronic pain as well as a reticence among healthcare providers to prescribe pain medications to Indigenous people on the presumption they are “drug-seeking” [5, 63]. While integrated healthcare is associated with improvements in mental health [64], calls to establish integrated pain management services are ongoing [65]. This is particularly important for IPWUD, who often experience intersectional drug user and race-based discrimination when seeking pain management.

A key finding arising from this study was that IPWUD whose providers were aware of traditional and cultural treatments were more likely to be involved in treatment decisions and to be comfortable with their provider or clinic than those who did not report provider awareness. Previous research has shown that receptivity to traditional and cultural treatments by culturally competent healthcare providers can improve trust and relationships between Indigenous communities and healthcare institutions, as well as improve health outcomes, especially in areas of substance use and mental health [20, 66, 67]. However, it is not clear from our findings whether the inclusion of traditional and cultural treatments is improving care, or whether those providers who possess the requisite knowledge, self-awareness, and skills to engage respectfully and relevantly with IPWUD are improving the care relationship more broadly, including through the inclusion of such treatments. Further, participants who reported provider awareness were experiencing more unstable housing or homelessness and chronic pain than their counterparts; perhaps traditional and cultural treatments are offered not as routine care but to those with the most complex healthcare needs. This finding may also be explained in part by an existing level of awareness among providers, given the large Indigenous population in the DTES [33] and the neighbourhood’s long history of advocacy for Indigenous health programming [68]. Altogether, these findings indicate that those who perceived their providers were aware of traditional and cultural treatments may have been experiencing a more positive client-provider relationship. This is congruent with previous research and Indigenous directives outlining the core components of culturally safe care, including mutual respect, accountability, cross-cultural communication, and shared decision-making [13, 69, 17]. Future research should continue to explore the work of Indigenous cultural safety champions who enact these core elements in their healthcare practice [70, 71] in order to inform future system reforms.

We also found that while a greater proportion of total respondents received care most frequently from a clinician (76%) instead of a social support worker, receiving care from a social support worker (i.e., counsellor, outreach worker, social worker, peer) was positively associated with perceived provider awareness of traditional and cultural treatments. This finding may point to a higher degree of comfort and trust with social support workers, particularly peers and outreach workers who have similar life experiences and social locations [72], and who can “humanize” healthcare [73]. Within this, it is important to note the history of harms perpetuated by Western medical systems against Indigenous Peoples, including in Indian Residential Schools, Indian Hospitals, and in medical experiments funded by the Canadian government and conducted on children [74, 38, 75], as well as contemporary widespread Indigenous-specific racism in BC’s health system [5]. Our finding is further evidence of the need for cultural safety training for healthcare professionals, but also of the value of social support workers in service provision. Lessons should be learned from Indigenous primary healthcare services, which have focused on recruiting and retaining Indigenous health workers and other holistic practitioners to provide a diverse range of care [76].

While there is a significant body of research documenting the health disparities between Indigenous and non-Indigenous people, much of the evidence describing the health status of Indigenous people is authored by non-Indigenous people, which poses risk to culturally safe contexts [74]; hence, the significance of Indigenous-specific data and research. More evaluative research of innovative cultural safety interventions is required [77, 52], as well as research that explores what successful integration of Western and Indigenous approaches in primary care looks like and how it impacts those receiving services [78]. On a practical level, health systems must continue to embed cultural safety in their policies and protocols while measuring its impact in order to achieve health equity [9]. It is recommended that Western health services simultaneously learn from ongoing innovations in Indigenous primary care to further transformative change [76, 79, 80].

### Limitations

This study has several limitations worth noting. First, by definition, we excluded those who did not access primary care, including those who accessed the emergency department, in the last 6 months. The most structurally marginalized IPWUD may not be attached to care, and therefore may not be captured here. Second, much harmful research on and about Indigenous Peoples has been conducted in Canada and elsewhere [81, 82], and subsequent mistrust of Western researchers and approaches may have affected participant recruitment. Last, some aspects of the regression analysis must be interpreted with caution – in particular, the finding that positive change in perceived provider awareness was observed over time, given the loss of participants to follow-up in the fourth and final survey round.

## Conclusion

Among a sample of Indigenous people who use drugs (IPWUD), we observed that a majority felt that their healthcare provider was aware of traditional and cultural treatments. We observed high rates of comfort with provider or clinic and accessing healthcare in one place, as well as associations between perceived provider awareness and client involvement in treatment decisions (always or most or some of the time), client comfort with provider or clinic, and service provision by a social support worker. This is the first study, to our knowledge, to examine the prevalence and correlates of perceived provider awareness of traditional and cultural treatments among IPWUD. Our findings suggest that despite a high level of perceived awareness in Vancouver's Downtown Eastside, broader Indigenous cultural safety implementation and accountability mechanisms are required to advance culturally safe care for IPWUD, who bear a disproportionate burden of substance use harms.

## Data Availability

All data generated during this study are included in this published article.

## Abbreviations

PWUD: People who use drugs
IPWUD: Indigenous people who use drugs
FNHA: First Nations Health Authority
VCH: Vancouver Coastal Health
DTES: Downtown Eastside
DTES-2GS: Downtown Eastside Second Generation Strategy
VIDUS: Vancouver Injection Drug Users Study
ACCESS: AIDS Care Cohort to Evaluate Exposure to Survival Services
WAHRS: Western Aboriginal Harm Reduction Society
